# Supplementary material for: The growth benefits and toxicity of quinone biosynthesis are balanced by a dual regulatory mechanism and substrate limitations
Source: mBio. 2025 Aug 11;16(9):e00887-25. doi: 10.1128/mbio.00887-25 (PMC12421875; doi:10.1128/mbio.00887-25)
Supplement: Supplemental material — Supporting figures, text, and tables. [file mbio.00887-25-s0001.pdf]

SUPPLEMENTARY MATERIAL FOR

The growth benefits and toxicity of quinone biosynthesis are balanced by a dual regulatory mechanism and substrate limitations

Siliang Li[1†], Jiangguo Zhang[2†], Caroline M. Ajo-Franklin[1,2,3,4*], Oleg A. Igoshin[1,2,4,5,6*]

[1]Department of BioSciences, Rice University, Houston, Texas.
[2]Department of Bioengineering, Rice University, Houston, Texas.
[3]Department of Chemical and Biomolecular Engineering, Rice University, Houston, Texas.
[4]Rice Synthetic Biology Institute, Rice University, Houston, Texas.
[5]Department of Chemistry, Rice University, Houston, Texas.
[6]Center for Theoretical Biological Physics, Rice University, Houston, Texas.

[†]These authors contributed equally

*Co-corresponding authors (these authors supervised this work jointly and equally): Caroline M. Ajo-Franklin, Oleg A. Igoshin

Corresponding authors' emails: cajo-franklin@rice.edu, igoshin@rice.edu

This PDF includes:

Supplementary Figures 2
Supplementary Texts 7
Supplementary Tables 8
Supplementary References 11

Supplementary Figures

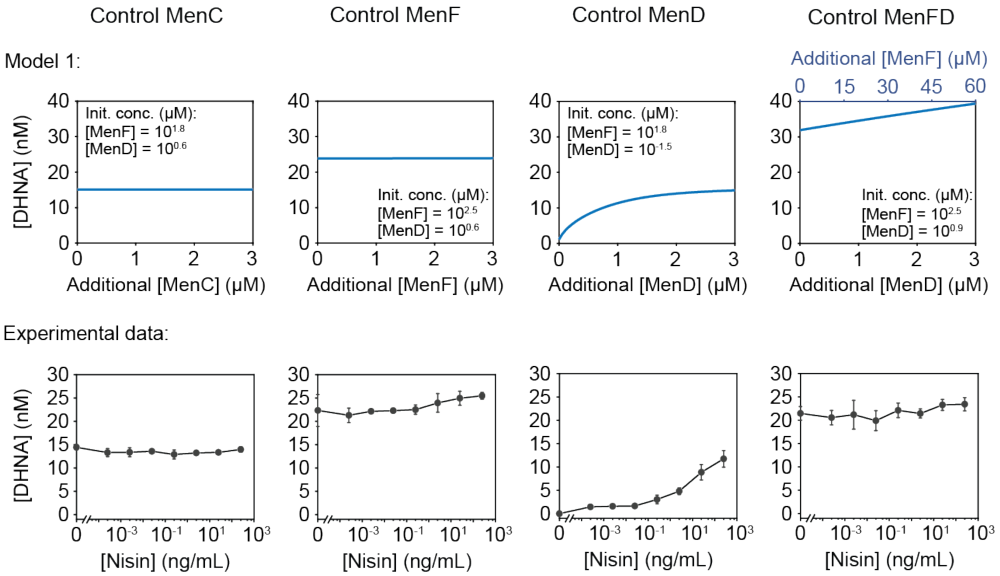

Figure S1: Model 1 correctly predicts DHNA concentrations in response to the perturbations of MenC, MenF, and MenD, but fails when applied to MenFD. The top row represents the predicted changes in DHNA concentrations in response to perturbations of MenC, MenF, and MenD, and MenFD, and the bottom row shows the experimental results. All experimental data represent mean ± 1 s.d. of n=3 biological replicates.

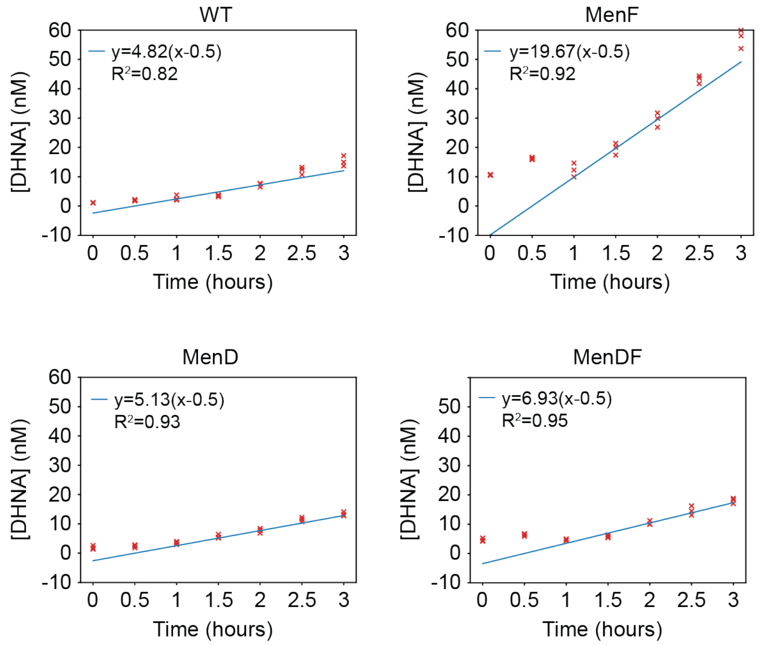

Figure S2: Determination of extracellular DHNA accumulation rate. The supernatants of *L. lactis* were collected at the indicated time points, and the extracellular DHNA concentrations were determined using the EET-based biosensing system. For calculating the DHNA accumulation rate, the linear regression function y=a(x-0.5) was fitted to the experimental data (mean of the three biological replicates) from 1 to 3 hours. The 0 and 0.5 hours data were omitted because we hypothesized that the DHNA outflux had not reached its steady-state rate within 0.5 hours. The MenF, MenD, and MenDF expressions were induced with 25 ng/mL nisin.

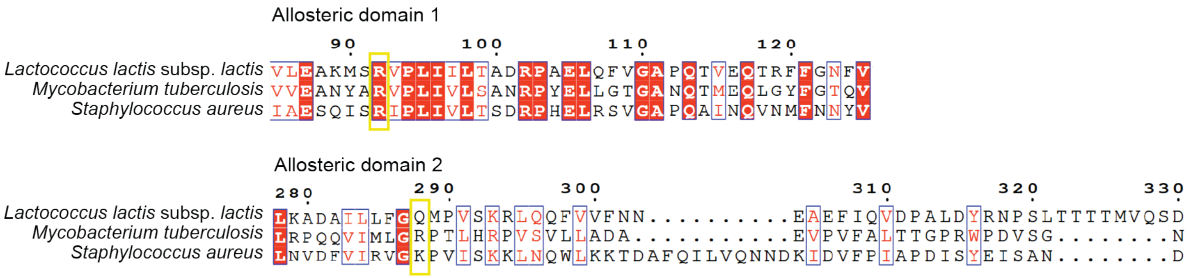

Figure S3: MenD allosteric site analysis. To see if *L. lactis* MenD possesses the key residues required for allosteric inhibition, we performed multiple sequence alignment comparing MenD sequences from *L. lactis*, *M. tuberculosis*[1], and *S. aureus*[2]. We found that a key residue previously validated in the DHNA-binding allosteric domain 1, Arg92 (highlighted by the top yellow square), is conserved across all three species. However, another potential residue for DHNA-binding in the allosteric domain 2 (the bottom yellow square) varies: it appears to be Gln288 in *L. lactis*, but Arg and Lys in *M. tuberculosis* and *S.* aureus, respectively. This low conservation of domain 2 residues is consistent with observations in other species[1]. This analysis suggests that *L. lactis* MenD could possess the allosteric inhibition site, but the DHNA-binding amino acids might differ.

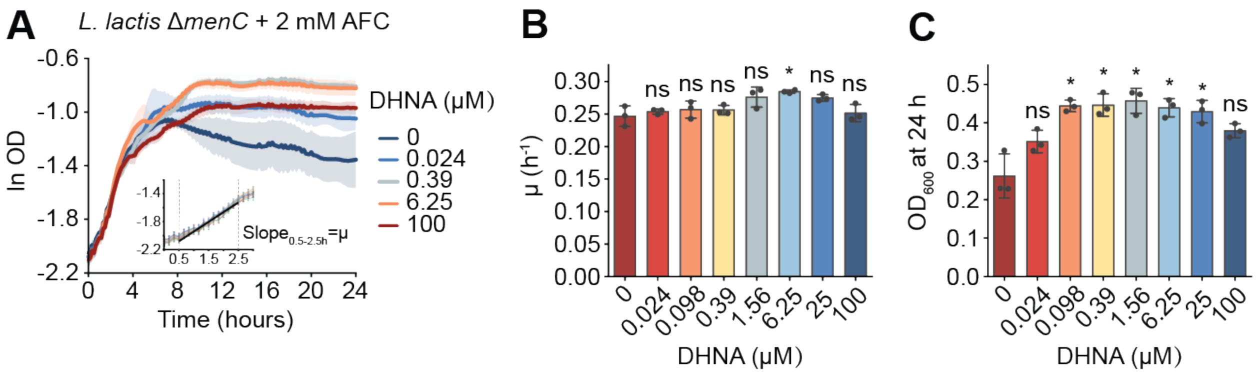

Figure S4: Exogenous DHNA rescues growth benefits in the Δ*menC* mutant. (A) Growth of *L. lactis* Δ*menC* mutant in the presence of varying concentrations of exogenous DHNA and 2 mM ammonium ferric citrate (AFC) under anaerobic conditions. For visual clarity, only 5 of the 8 tested DHNA concentrations are shown. The inset shows how the specific growth rate (μ) was calculated from the slope of a linear regression of the growth curve between 0.5 and 2.5 hours. **(B)** Specific growth rate under different exogenous DHNA concentrations. **(C)** Final cell density (OD_{600}) at 24 hours. P-values were determined by one-way ANOVA with Tukey's test. P-values comparing each condition to the DHNA-null control (0 μM) are shown. *p < 0.05; ns, not significant.

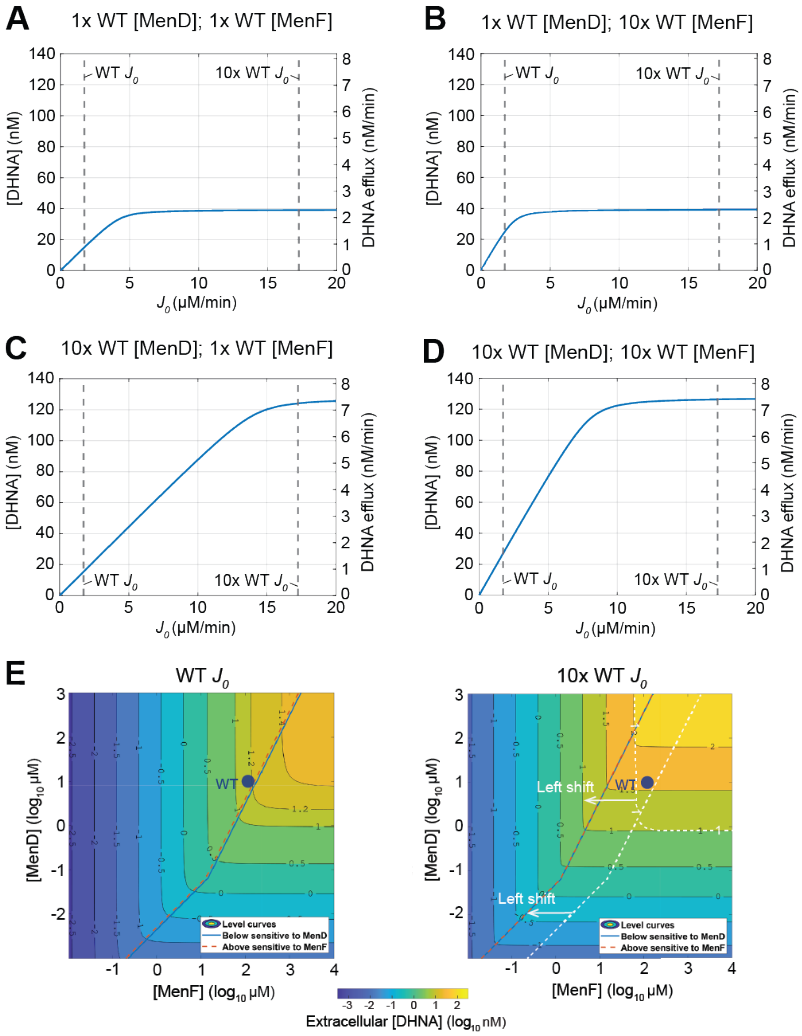

Figure S5: Effects of chorismate production rate (J_0) and MenD/MenF expression levels on extracellular DHNA concentrations and efflux. (**A–D**) Extracellular DHNA concentration after 2 hours (left axis) and DHNA efflux rate (right axis) plotted against varying chorismate production rate (J_0). The left dashed line indicates wild-type (WT) J_0, and the right dashed line indicates 10× WT J_0. Conditions shown: (**A**) Wild-type levels of MenD and MenF; (**B**) 10-fold overexpression of MenF only; (**C**) 10-fold overexpression of MenD only; (**D**) 10-fold overexpression of both MenD and MenF. (**E**) Comparison of DHNA-MenD-MenF level curves at wild-type (WT J_0) or 10-fold increased chorismate production rate (10×WT J_0). Increased J_0 shifts the level curves leftward, elevating the maximum limit of DHNA production.

Supplementary Texts

Related to Fig. S4

To evaluate if exogenous DHNA can rescue the growth benefits of *L. lactis* Δ*menC* mutant, we grew the mutant for 24 hours in the presence of varying concentrations of DHNA and 2 mM ammonium ferric citrate under anaerobic conditions (**Fig. S4A**). Compared to the DHNA null control, the specific growth rate during exponential growth phase was significantly enhanced with 6.25 µM exogenous DHNA (**Fig. S4B**), and the final cell density (OD_{600} at 24 h) increased gradually with 24 nM to 1.56 µM DHNA (**Fig. S4C**). These indicate that exogenous DHNA can rescue the growth benefits of the Δ*menC* mutant. Interestingly, while endogenously synthesized DHNA promotes growth during early exponential growth phase (**Fig. 4B**), the benefit of exogenous DHNA appears at late exponential or stationary growth phases. This delay may reflect the need for cellular uptake and processing of exogenous DHNA (e.g., conversion to DMK or MK) before exerting its beneficial effects. However, exogenous DHNA concentration above 6.25 µM started to impair both the specific growth rate and the final cell density, suggesting toxicity at high levels. This toxic threshold is higher than that observed in **Fig. 4A** (above 390 nM), possibly due to increased DHNA tolerance in the Δ*menC* background, or the presence of ammonium ferric citrate mitigates the DHNA toxicity. Together, these results demonstrate that exogenous DHNA can rescue the growth benefits of the Δ*menC* mutant, but excessive DHNA exhibits toxicity, providing another evidence that *L. lactis* must tightly regulate DHNA biosynthesis.

Related to Fig. S5

We performed additional simulations to show how extracellular DHNA concentration and efflux rate respond to increasing levels of chorismate and MenF/D. When MenF/D remain at the wild-type levels, a 10-fold increase in chorismate production rate (J_0) leads to a ~2- to 2.5-fold increase in DHNA level and efflux rate, when compared to the wild-type conditions (**Fig. S5A**). Overexpression of MenD alone (**Fig. S5C**) or MenD together with MenF (**Fig. S5D**), but not MenF alone (**Fig. S5B**), by 10-fold can further increase the maximum DHNA level and efflux by almost 8-fold. Why MenF overexpression alone was insufficient to increase at higher J_0? Under wild-type J_0, the system operates under a regime where MenF can be flux-limiting, making MenF overexpression effective to increase DHNA levels and efflux. However, under a 10-fold increased J_0, the concentration of chorismate, [A], is high enough so that, in agreement with **Equation 21**, it is the concentration of MenD rather than MenF that is limiting the reaction flux into DHNA.

To further illustrate these phenomena, we analyzed the impact of 10-fold increase of J_0 on the DHNA level curves in **Model 2** and observed two main effects (**Fig. S5E**): 1) Increased J_0 elevates the maximum limit of extracellular DHNA concentration ($[DHNA]_{ex,2h}^{max}$) and DHNA efflux, in agreement with **Equation 29**; 2) Increased J_0 shifts the entire level curves leftward, placing the wild-type MenF-to-MenD ratio below the phase boundary, a region where MenD, but not MenF, is the flux-limiting step. This again explains why overexpression of MenF under 10-fold increased J_0 could not elevate DHNA levels. Collectively, these findings demonstrate that increasing chorismate production can elevate DHNA levels and efflux, with further elevation possible through overexpression of MenD alone or MenD together with MenF.

Supplementary Tables

Table S1. Strains used in this study

Species	Genotype	Plasmid	Source
L. plantarum	NCIMB8826 *ΔdmkA Δndh1*		[3]
L. plantarum	NCIMB8826 *ΔdmkA Δndh1 Δndh2*		[3]
L. lactis subsp. *lactis*	KF147 Δ*noxAB* Δ*menA*		This study
L. lactis subsp. *lactis*	KF147 Δ*menC* Δ*noxAB* Δ*menA*		This study
L. lactis subsp. *lactis*	KF147 Δ*menC* Δ*noxAB* Δ*menA*	pECGMC3-P32_*menC*	This study
L. lactis subsp. *lactis*	KF147 Δ*menC* Δ*noxAB* Δ*menA*	pECGMC3-PnisA_strongRBS_*mCherry-menC*	This study
L. lactis subsp. *lactis*	KF147 Δ*menC* Δ*noxAB* Δ*menA*	pECGMC3-PnisA_weakRBS_*mCherry-menC*	This study
L. lactis subsp. *lactis*	KF147 Δ*menC* Δ*noxAB* Δ*menA*	pECGMC3-PnisA_weakRBS_*menC*	This study
L. lactis subsp. *lactis*	KF147 Δ*menC* Δ*noxAB* Δ*menA* Δ*menF*	pECGMC3-P32_*menC*-PnisA_strongRBS_*mCherry-menF*	This study
L. lactis subsp. *lactis*	KF147 Δ*menC* Δ*noxAB* Δ*menA* Δ*menF*	pECGMC3-P32_*menC*-PnisA_weakRBS_*mCherry-menF*	This study
L. lactis subsp. *lactis*	KF147 Δ*menC* Δ*noxAB* Δ*menA* Δ*menF*	pECGMC3-P32_*menC*-PnisA_ weakRBS _*menF*	This study
L. lactis subsp. *lactis*	KF147 Δ*menC* Δ*noxAB* Δ*menA* Δ*menD*	pECGMC3-P32_*menC*_PnisA_ strongRBS _*mCherry-menD*	This study
L. lactis subsp. *lactis*	KF147 Δ*menC* Δ*noxAB* Δ*menA* Δ*menD*	pECGMC3-P32_*menC*_PnisA_ weakRBS _*mCherry-menD*	This study
L. lactis subsp. *lactis*	KF147 Δ*menC* Δ*noxAB* Δ*menA* Δ*menD*	pECGMC3-P32_*menC*_PnisA_weakRBS _*menD*	This study
L. lactis subsp. *lactis*	KF147 Δ*menC* Δ*noxAB* Δ*menA* Δ*menFD*	pECGMC3-P32_*menC*_PnisA_ weakRBS _*menF*_nativeRBS_*menD*	This study
L. lactis subsp. *lactis*	KF147 Δ*menC* Δ*noxAB* Δ*menA* Δ*menFD*	pECGMC3-P32_*menC*-PnisA_ weakRBS _*menD*_nativeRBS_*menF*	This study

Table S2. Recipe of mannitol-MRS (mMRS)

Component	Final concentration (g/L)
Protease Peptone #3	10
Tween80	1
Mannitol	10
Yeast Extract	5
Potassium Phosphate dibasic	2
Sodium acetate trihydrate	8.3
ammonium citrate tribasic	2.15
Magnesium sulfate anhydrous	0.1
Manganese sulfate monohydrate	0.05

Table S3. Recipe of mannitol-chemically defined medium (mCDM)

Component	Final concentration (g/L)
**Buffers and salts**	
MOPS (3-(N-morpholino)propanesulfonic acid)	8.371
K_2HPO_4	0.871
NH_4Cl	1.070
Na_2SO_4	1.420
**Carbon source**	
Mannitol	10
**Metals**	
$MgCl_2$ * $6H_2O$	0.203
$MnCl_2$ * $4H_2O$	0.01
$FeSO_4$ * $7H_2O$	0.014
**Amino acids**	
Casamino acids	1.500
Cysteine-HCl * H_2O	0.145
Tryptophan	0.050
**Wolfe's Vitamins**	
Pyridoxine HCl	0.001
Thiamine HCl	0.0005
Riboflavin	0.0005
Nicotinic acid	0.0005
Calcium D-(+)-pantothenate	0.0005
p-Aminobenzoic acid	0.0005
Thioctic acid (α-Lipoic acid)	0.0005
Biotin	0.0002
Folic acid	0.0002
Vitamin B12	0.00001
**Wolfe's Minerals**	
Nitrilotriacetic acid (NTA)	0.15
$MgSO_4$ * $7H_2O$	0.3
$MnSO_4$ * H_2O	0.05
NaCl	0.1
$FeSO_4$ * $7H_2O$	0.01
$CoCl_2$ * $6H_2O$	0.01
$CaCl_2$	0.01
$ZnSO_4$ * $7H_2O$	0.01
$CuSO_4$ * $5H_2O$	0.001
$AlK(SO)_4$ * $12H_2O$	0.001
H_3BO_3	0.001
Na_2MoO_4 * $2H_2O$	0.001

Supplementary References

1. Bashiri, G., Nigon, L.V., Jirgis, E.N.M., Ho, N.A.T., Stanborough, T., Dawes, S.S., Baker, E.N., Bulloch, E.M.M., and Johnston, J.M. (2020). Allosteric regulation of menaquinone (vitamin K2) biosynthesis in the human pathogen Mycobacterium tuberculosis. J Biol Chem *295*, 3759–3770. https://doi.org/10.1074/jbc.RA119.012158.

2. Stanborough, T., Ho, N.A.T., Bulloch, E.M.M., Bashiri, G., Dawes, S.S., Akazong, E.W., Titterington, J., Allison, T.M., Jiao, W., and Johnston, J.M. (2023). Allosteric inhibition of Staphylococcus aureus MenD by 1,4-dihydroxy naphthoic acid: a feedback inhibition mechanism of the menaquinone biosynthesis pathway. Philosophical Transactions of the Royal Society B: Biological Sciences *378*, 20220035. https://doi.org/10.1098/rstb.2022.0035.

3. Li, S., De Groote Tavares, C., Tolar, J.G., and Ajo-Franklin, C.M. (2024). Selective bioelectronic sensing of pharmacologically relevant quinones using extracellular electron transfer in Lactiplantibacillus plantarum. Biosensors and Bioelectronics *243*, 115762. https://doi.org/10.1016/j.bios.2023.115762.
